# A brainstem circuit for phonation and volume control in mice

**DOI:** 10.1038/s41593-023-01478-2

**Published:** 2023-11-23

**Authors:** Avin Veerakumar, Joshua P. Head, Mark A. Krasnow

**Affiliations:** 1 Department of Biochemistry and Howard Hughes Medical Institute, Stanford University School of Medicine, Stanford, CA USA; 2 Department of Bioengineering, Stanford University, Stanford, CA USA; 3 Medical Scientist Training Program, Stanford University School of Medicine, Stanford, CA USA; 4 Neurosciences Program, Stanford University, Stanford, CA USA

**Keywords:** Neural circuits, Molecular neuroscience, Motor control, Social behaviour

## Abstract

Mammalian vocalizations are critical for communication and are produced through the process of phonation, in which expiratory muscles force air through the tensed vocal folds of the larynx, which vibrate to produce sound. Despite the importance of phonation, the motor circuits in the brain that control it remain poorly understood. In this study, we identified a subpopulation of ~160 neuropeptide precursor *Nts* (neurotensin)-expressing neurons in the mouse brainstem nucleus retroambiguus (RAm) that are robustly activated during both neonatal isolation cries and adult social vocalizations. The activity of these neurons is necessary and sufficient for vocalization and bidirectionally controls sound volume. RAm *Nts* neurons project to all brainstem and spinal cord motor centers involved in phonation and activate laryngeal and expiratory muscles essential for phonation and volume control. Thus, RAm *Nts* neurons form the core of a brain circuit for making sound and controlling its volume, which are two foundations of vocal communication.

## Abstract (web summary)

The authors identify a cluster of ~160 peptidergic neurons in the mouse brainstem whose activity is necessary and sufficient for producing sound and controlling sound volume. These neurons form the final common pathway for vocalization.

## Main

Animals communicate through a range of vocalizations. Many of these are innate, such as a laugh, a baby’s cry, a dog’s bark or a rodent’s distinct calls for its mother or a mate. A small number of bird and mammalian species can also produce learned vocalizations, such as human speech^1^. All types of vocalizations in mammals, both innate and learned, are generated by the larynx, an airway structure containing two vocal folds that, when tensed, vibrate and generate sound as air is moved across them. The vocal folds are brought together (adducted) and tensed by laryngeal muscles, and air is moved across them by abdominal muscles, which generate expiratory force during vocalization. This process of laryngeal sound production (phonation) is central to all mammalian vocalizations, including speech. As sound is produced, fine modulations of expiratory force, laryngeal muscle tension and timing of muscle contractions generate acoustic features, such as loudness (volume), pitch, syllable structure and syntax, which convey meaning to the listener. Despite the importance of phonation, little is known about the neuronal cell types and motor circuits controlling phonation or individual acoustic features.

**Extended Data Fig. 1:**
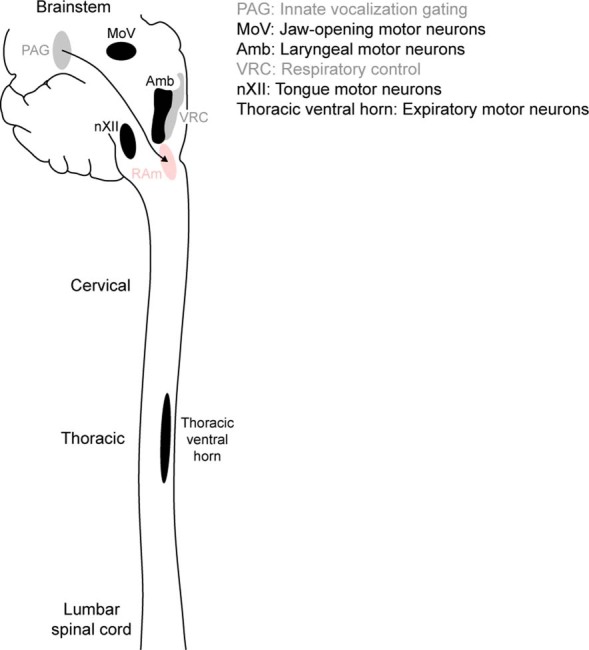
Schematic of brainstem regions involved in innate vocalization. Innate vocalizations are generated (gated) by the midbrain periaqueductal gray (PAG) in the brainstem, which sends dense axonal projections to the nucleus retroambiguus (RAm, red) in the caudal medulla. Motor neuron pools (black) innervate the jaw opening muscles (trigeminal motor nucleus, MoV), laryngeal muscles (nucleus ambiguus, Amb), tongue muscles (hypoglossal motor nucleus, nXII), and abdominal expiratory muscles (thoracic ventral horn), which are all involved in vocalization. The ventral respiratory column (VRC, gray) contains inspiratory and expiratory control centers. RAm is hypothesized to control these distant motor pools and breathing centers to generate vocalization.

The motor neurons controlling the laryngeal muscles reside in the nucleus ambiguus (Amb) in the medulla, whereas motor neurons controlling abdominal expiratory muscles reside in the thoracic spinal cord (Extended Data Fig. 1). Motor centers controlling other key aspects of phonation are located across the pons and medulla, in the trigeminal motor nucleus (mouth opening), in the ventral respiratory column (control of inspiration and expiration) and in the hypoglossal nucleus (tongue positioning). How are these disparate and distant motor output centers intricately coordinated during vocalization? Classical anatomical and physiological studies in cats and primates identified the midbrain periaqueductal gray (PAG) as a key center controlling innate vocalization^2^. PAG stimulation results in natural-sounding vocalizations, whereas PAG lesions cause mutism^2^. PAG vocalization-active neurons innervate many targets in the lower brainstem, including the nucleus retroambiguus (RAm)^3^, located caudal to Amb. Medullary transections at the level of RAm abolish PAG-driven vocalization in decerebrate cats^4^, suggesting that RAm is a critical relay node for innate vocalization. Chemical stimulation of RAm with a glutamate analog produces artificial-sounding vocalizations^4^, and bulk anterograde tracing studies from RAm found projections to the brainstem and spinal cord regions implicated in phonation^5^. Although these classical studies indicate an important role for RAm in vocalization, the neuronal cell types in this region and their circuitry and specific contributions to vocalization are unknown.

In this study, we employed neural activity mapping, optogenetics, behavioral experiments and neuroanatomical tracing in mice to genetically identify and characterize a RAm vocalization cell type. Although most mammals produce vocalizations that are audible to humans (<20 kHz), rodents can also produce ultrasonic vocalizations (>20 kHz) via a laryngeal whistle mechanism^6^. Because both audible^7^ and ultrasonic^8^ vocalizations are produced by using expiratory force to push air through the adducted larynx, we will use the term ‘phonation’ when referring to either human-audible or ultrasonic sound production. Here we show that a subpopulation of ~160 vocalization-activated RAm neurons express the neuropeptide precursor gene *Nts* (neurotensin). RAm *Nts* neurons are an excitatory subpopulation activated by both neonatal isolation cries and adult social vocalizations. Genetically targeted gain-of-function and loss-of-function studies show that RAm *Nts* neurons are necessary for adult social vocalization, sufficient for phonation in the audible and ultrasonic ranges, and their neural activity level determines the volume of the produced sound. RAm *Nts* neurons project to all motor pools involved in phonation and engage key laryngeal and expiratory muscles when activated. Our studies indicate that RAm *Nts* neurons define a critical brain circuit for phonation and volume control.

## Results

### *Nts* marks a subset of vocalization-activated neurons in RAm

**Fig. 1:**
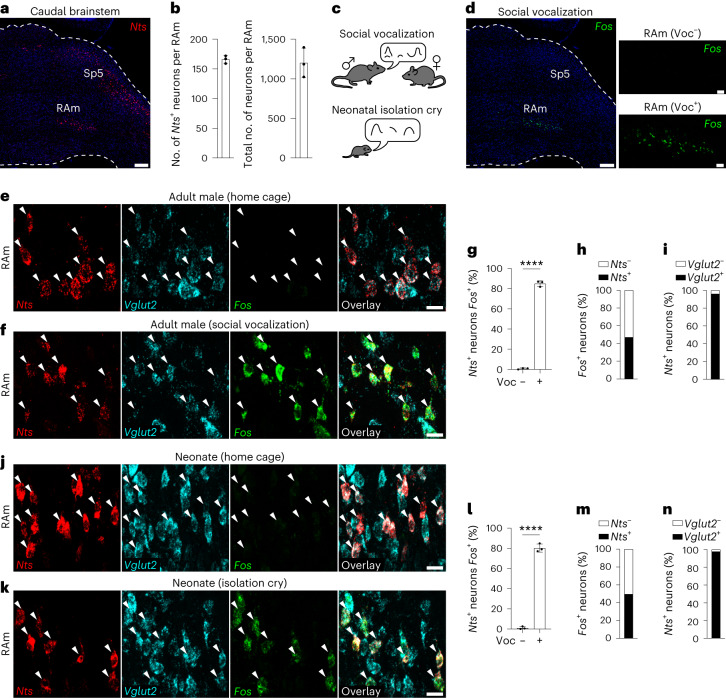
*Nts* marks a vocalization-activated RAm subpopulation. **a**, Caudal brainstem sagittal section of adult mouse with *Nts* mRNA labeled by smFISH (red). Note *Nts* expression in RAm and spinal trigeminal nucleus (Sp5). Scale bar, 200 µm. **b**, Left, number of *Nts*^+^ neurons in RAm (*n* = 3 mice). Right, number of neurons in RAm (*n* = 3 mice). **c**, Top, adult social vocalization. A female mouse placed with a male drives ultrasonic vocalizations by the male. Bottom, neonatal isolation cry. Removing a neonatal mouse from home cage drives ultrasonic isolation cries. **d**, Left, *Fos* mRNA labeling by smFISH (green, neural activity marker) in an adult male that vocalized to a female for 90 min. Note *Fos* expression restricted to RAm (magnified in right lower panel). Right upper panel, *Fos* labeling in RAm of adult male home cage control mouse that did not vocalize. Left scale bar, 200 µm. Right panel scale bars, 50 µm. **e**, smFISH labeling for *Nts*, *Vglut2* and *Fos* in RAm of an adult male home cage control mouse. Note all RAm *Nts*^+^ neurons (arrowheads) express *Vglut2* but do not express *Fos*. Scale bar, 15 µm. **f**, Identical smFISH labeling in RAm of adult male mouse that vocalized to a female. Note induction of *Fos* in RAm *Nts*^+^ neurons. Scale bar, 15 µm. **g**, Percent of RAm *Nts*^+^ neurons that express *Fos* in adult male mice under home cage control (Voc^−^) and vocalization (Voc^+^) conditions (*P* = 9 × 10^−7^, *n* = 3 mice per condition and 739 total scored *Nts*^+^ neurons). **h**, Percent of RAm *Fos*^+^ neurons in vocalizing adult male mice that also express *Nts* (*n* = 3 mice and 710 total scored *Fos*^+^ neurons). **i**, Percent of RAm *Nts*^+^ neurons that express *Vglut2* in adult male mice (*n* = 3 mice and 739 total scored *Nts*^+^ neurons). **j**–**n**, Experimental scheme as in **c**–**i** except performed in neonatal mice that emitted isolation cries or home cage controls (*P* = 6 × 10^−6^, *n* = 3 mice per condition, 1,164 total scored *Nts*^+^ neurons and 1,115 total scored *Fos*^+^ neurons). Scale bars, 15 µm. Data are shown as mean ± s.d. ****P < 0.0001 by unpaired two-tailed *t*-test. Source data

To identify genetic markers of vocalization neurons, we searched the Allen Mouse Brain Atlas^9^ for genes selectively expressed in RAm. One of the identified genes was *Nts*, which encodes the preproprotein for neuropeptides neurotensin and neuromedin N (ref. ^10^). Single-molecule fluorescence in situ hybridization (smFISH) (Fig. 1a) confirmed *Nts* expression in RAm as well as in the spinal trigeminal nucleus and a sparse population of motor neurons in the Amb^9^. Cell counts showed ~160 *Nts*-expressing neurons in RAm, with the *Nts*-expressing neurons clustered but intermingled with ~1,100 non-expressing neurons (Fig. 1b). To determine if RAm *Nts* neurons are activated during vocalization, we placed adult male mice with a female mouse to induce social vocalizations^3^ (Fig. 1c, top) and then examined activity of *Nts* and other RAm neurons with the neural activity marker *Fos*, allowing 90 min for *Fos* transcripts to accumulate. There were few, if any, *Fos*-expressing (active) neurons in RAm or surrounding regions in home cage male control mice, but there was robust induction of *Fos* in the RAm of vocalizing male mice (Fig. 1d). Probing of RAm sections for both *Nts* and *Fos* showed that RAm *Nts* neurons are activated during vocalization: ~80% of RAm *Nts*^+^ neurons expressed *Fos* in the vocalizing mice, whereas less than 1% expressed *Fos* in control mice (Fig. 1e–g). *Nts*^+^ neurons comprised ~45% of the *Fos*^+^ neurons in RAm, indicating that there is at least one other population of vocalization-activated RAm neurons, which is intermingled with and surrounds the *Nts* population (Fig. 1f,h). Over 95% of RAm *Nts* neurons expressed the excitatory marker *Vglut2* (vesicular glutamate transporter 2) (Fig. 1e,f,i).

To determine if RAm *Nts*^+^ neurons are activated during other types of vocalization, we examined the activity of *Nts* neurons in neonatal mice induced to produce isolation calls by removal from their home cage litter^11^ (Fig. 1c, bottom). In control pups that remained in their home cage, only ~1% of RAm *Nts* neurons expressed *Fos* (Fig. 1j,l). However, in vocalizing pups removed from the home cage, ~80% of RAm *Nts* neurons expressed *Fos* (Fig. 1k,l). As during adult social vocalizations, *Nts* neurons comprised ~50% of the total *Fos*^+^ neurons in RAm (Fig. 1m), and more than 95% of the *Nts* neurons were *Vglut2*^*+*^ (Fig. 1n).

**Extended Data Fig. 2:**
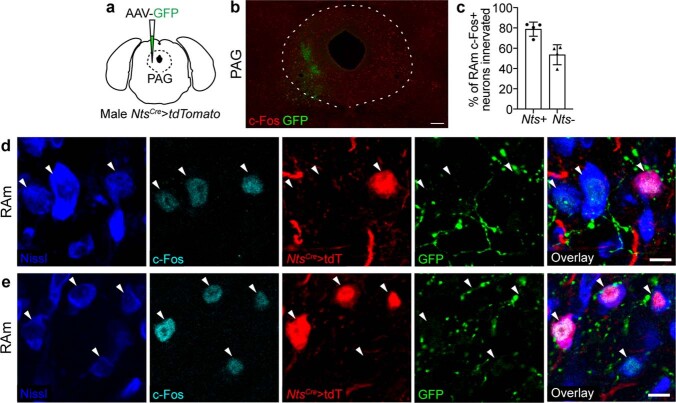
PAG innervation of RAm *Nts+* and *Nts-* neurons. **a**, Experimental schematic: AAV-GFP was injected into the caudolateral PAG of male mice to label projections of vocalization-gating neurons, and 3 weeks later male mice were induced to vocalize towards a female for 90 minutes before GFP and c-Fos immunostaining. **b**, Immunostaining for GFP and c-Fos in coronal brain slice containing caudal PAG following injection of AAV-GFP into left caudolateral PAG and subsequent vocalization induction. Note c-Fos expression across the PAG, and injection site targeting the left caudolateral PAG. Scale bar, 200 µm. **c**, Quantification of PAG innervation of RAm c-Fos+ neurons (*n* = 4 mice). **d**, Representative immunostaining image of RAm showing three c-Fos+ neurons (arrowheads) also labeled with Nissl (neuron-filling stain), tdTomato (*Nts*^*Cre*^+ neurons), and GFP (PAG innervation). Note right *Nts*^*Cre*^ + /c-Fos+ neuron receiving PAG innervation (puncta), and two left *Nts*^*Cre*^-/c-Fos+ neurons also receiving PAG innervation. Scale bar, 10 µm. **e**, Additional representative immunostaining image showing four c-Fos+ neurons (arrowheads). Note three top neurons are *Nts*^*Cre*^ + /c-Fos+ and receive PAG innervation, whereas bottom-right neuron is *Nts*^*Cre*^-/c-Fos+ and does not receive PAG innervation. Scale bar, 10 µm. All data shown as mean ± S.D. Source data

To test whether RAm *Nts* neurons are innervated by PAG neurons, we GFP labeled the caudolateral PAG with AAV-GFP in *Nts*^*Cre*^*:Ai14* adult male mice in which *Nts* neurons are labeled with tdTomato (Extended Data Fig. 2a,b). The adult male mice were induced to vocalize as above, and RAm was co-stained for c-Fos along with GFP and tdTomato. RAm *Nts* neurons (~80%) were innervated by PAG, showing that they receive direct input from this vocalization gating region (Extended Data Fig. 2c–e). c-Fos^+^*Nts*^−^ neurons (~50%) were also innervated by PAG, showing that at least one other vocalization-active neuronal population (c-Fos^+^*Nts*^−^) in RAm is also directly engaged by PAG.

We conclude that *Nts* neurons are an excitatory subpopulation of RAm neurons that are robustly activated during both neonatal isolation cries and adult social vocalization, and they are directly innervated by PAG neurons.

### Ablation of RAm *Nts* neurons abolishes social vocalizations

**Fig. 2:**
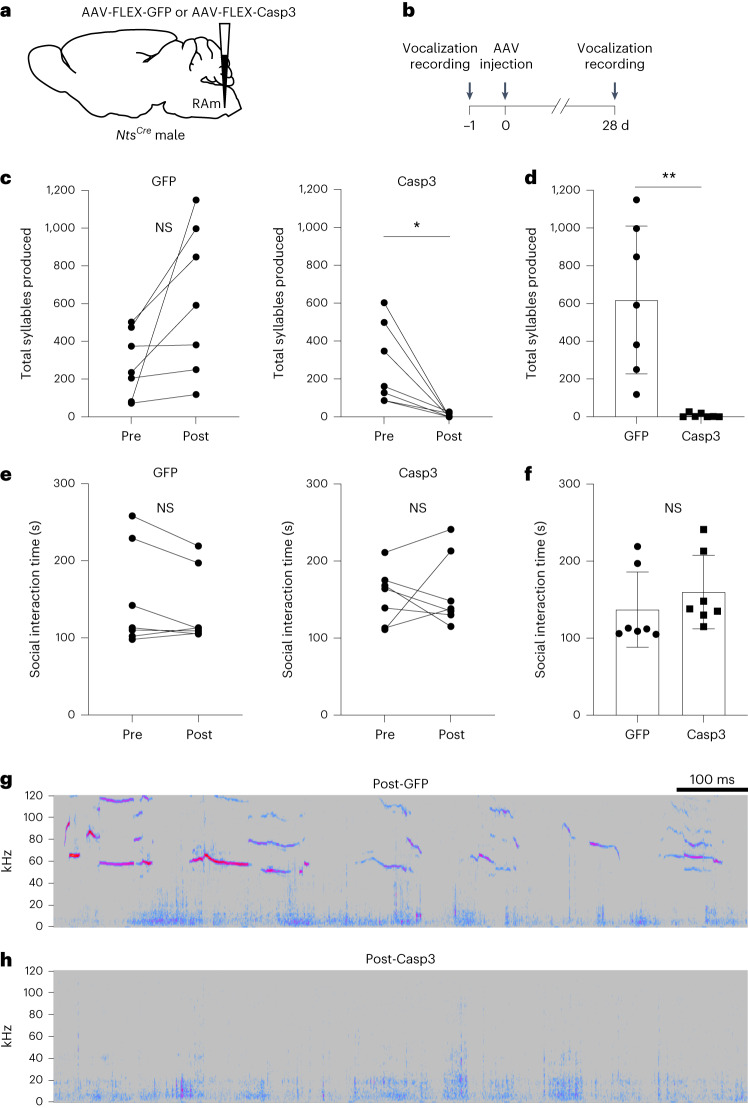
Effect of RAm *Nts* neuron ablation on social vocalizations. **a**, RAm *Nts* neurons were ablated by injecting Cre-dependent AAV encoding GFP (AAV-FLEX-GFP, mock ablation control) or apoptosis inducer Casp3 (AAV-FLEX-Casp3, ablation condition) in adult male *Nts*^*Cre*^ mice. **b**, Experimental paradigm. Female-induced vocalizations from male *Nts*^*Cre*^ mice were recorded in a 5-min trial. One day later (day 0), AAV encoding Cre-dependent GFP or Casp3 was bilaterally injected into RAm of male *Nts*^*Cre*^ mice. After 28 d (to allow protein expression and cell ablation), the *Nts*^*Cre*^ mice were induced to vocalize during an identical 5-min trial with a female. **c**, Syllables produced during 5-min female encounters before (Pre) and after (Post) injection of Cre-dependent GFP (left) or Casp3 (right) (*n* = 7 mice per group). Note abolishment of vocalizations in Casp3 (ablation) (*P* = 0.018) condition. Trend toward increase in syllables in GFP (control) condition (*P* = 0.053) could be due to increased motivation to vocalize after 28-d social isolation between trials. **d**, Total syllables produced after GFP or Casp3 expression in RAm *Nts* neurons (*P* = 0.0014, *n* = 7 mice per group). **e**, Social interaction time during 5-min female encounters before (Pre) and after (Post) injection of Cre-dependent GFP (left) or Casp3 (right) (*n* = 7 mice per group). **f**, Social interaction time after GFP or Casp3 expression in RAm *Nts* neurons (*P* = 0.4, *n* = 7 mice per group). **g**, Sonogram of female-induced ultrasonic vocalizations by control *Nts*^*Cre*^ mouse 28 d after AAV-FLEX-GFP injection into RAm. *y* axis, frequency (kHz). Warmer colors indicate increased sound amplitude at given frequency. Note complex ultrasonic (>20 kHz) syllables. Audible sounds (<20 kHz) are background noises from mouse movement. **h**, Sonogram of female-induced ultrasonic vocalizations emitted by an *Nts*^*Cre*^ mouse 28 d after AAV-FLEX-Casp3 injection into RAm. Note absence of ultrasonic vocalizations. Data are shown as mean ± s.d. *P < 0.05, **P < 0.01 and NS, not significant, by paired (Pre versus Post) or unpaired (GFP versus Casp3) two-tailed *t-*test. Mouse brain schematic here and those in Figs. 3, 4, 5 and 6 and Extended Data Figs. 1, 2 and 5 are reproduced from ref. ^33^. Source data

**Extended Data Fig. 3:**
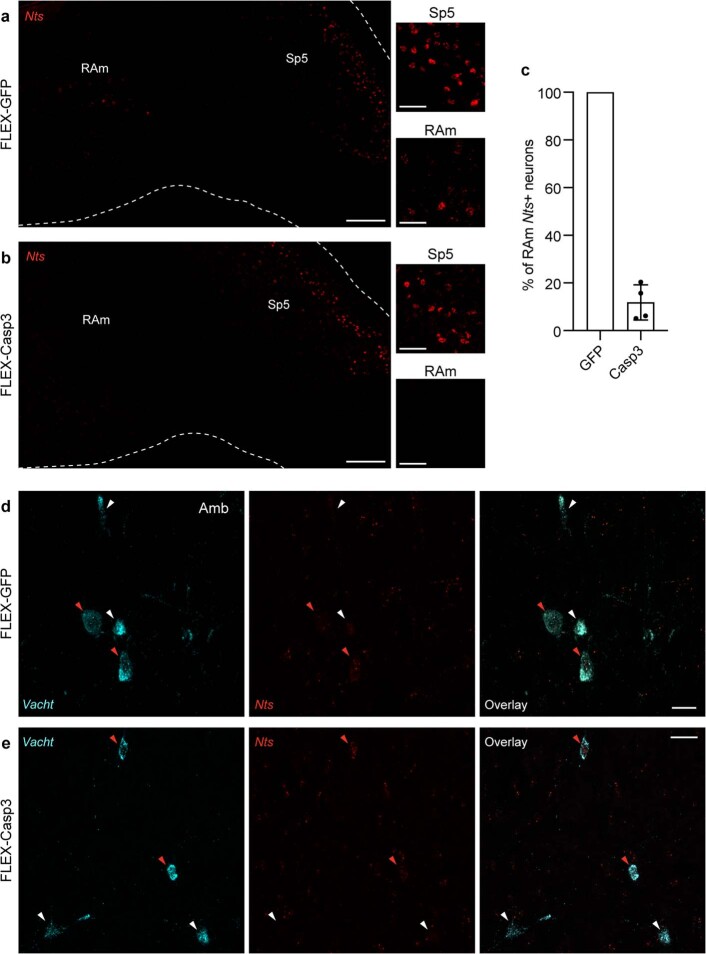
Casp3-mediated ablation of RAm *Nts* neurons. **a**, **b**, smFISH labeling of *Nts* in brainstem of *Nts*^*Cre*^ mice injected in RAm with AAV-FLEX-GFP (a, mock ablation) or AAV-FLEX-Casp3 (b, ablation condition) for RAm *Nts* neuron ablation experiments (see Fig. 2). Insets, magnified view of Sp5 and RAm regions. Note ablation of RAm *Nts*+ neurons in Casp3 mice with sparing of Sp5. Scale bars, 200 µm. Inset scale bars, 50 µm. **c**, Percent of RAm *Nts*+ neurons remaining following AAV-FLEX-Casp3 injection relative to GFP mock ablation controls. *n* = 868 total RAm *Nts* neurons scored in 4 mice per condition. **d**, **e**, smFISH of *Nts*+ subpopulation of Amb motor neurons in GFP (d) and Casp3 (e) conditions above. Red arrowheads, *Nts*+ Amb motor neurons, marked by expression of *Vacht* (vesicular acetylcholine transporter). White arrowheads, *Nts*- Amb motor neurons. Note sparing of Amb *Nts*+ neurons in Casp3 condition, indicating AAV8 approach was selective for RAm interneurons and avoided nearby motor neurons. Scale bars, 25 µm. All data shown as mean ± S.D. Source data

To test for function of RAm *Nts* neurons in vocalization, we genetically ablated them and examined the effect on social vocalizations. Adult male *Nts*^*Cre*^ mice were bilaterally injected in RAm with a Cre-dependent adeno-associated virus (AAV) vector encoding Caspase-3 (Flex-Casp3) (Fig. 2a,b), which causes Cre-dependent apoptosis^12^. To avoid targeting the sparse population of *Nts*-expressing motor neurons in the adjacent Amb, we used AAV serotype 8, which transduces interneurons but has poor tropism for motor neurons^13^. Comparing the number of residual RAm *Nts*^*+*^ neurons in Flex-Casp3-injected mice to that of Flex-GFP (mock ablation) controls indicated that this approach ablated nearly all (~90%) RAm *Nts* neurons, and ablation was specific for RAm *Nts* neurons (Extended Data Fig. 3). Comparison of social vocalizations in the same animals before and after RAm *Nts* ablation showed that their ablation almost completely abolished vocalization (Fig. 2c,d,h), whereas mock ablation (Flex-GFP injection) had no effect (Fig. 2c,d,g). The effect of ablation on vocalization was not due to decreased activity or interest in the female, because Flex-Casp3 mice spent the same amount of time interacting with the female (Fig. 2e,f) and in a similar manner (Supplementary Videos 1 and 2) before and after RAm *Nts* ablation and compared to Flex-GFP control mice. We conclude that RAm *Nts* neurons are required to produce adult male-to-female social vocalizations, and their role is specific for vocalization.

### Activation of RAm *Nts* neurons produces vocalization

**Fig. 3:**
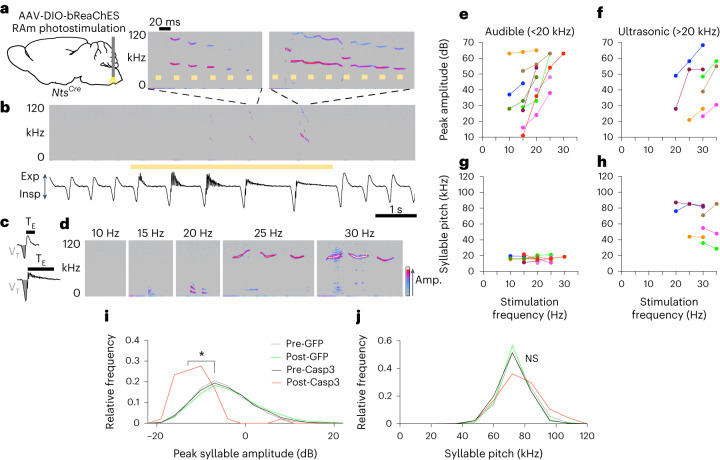
Optogenetic activation of RAm *Nts* neurons generates vocalizations and controls sound volume. **a**, RAm *Nts* neuron optogenetic stimulation by bilateral injection of Cre-dependent AAV encoding bReaChES in *Nts*^*Cre*^ mice, with laser light delivered via optical fiber. **b**, Respiratory airflow (bottom) and sonogram (top) during optogenetic stimulation of RAm *Nts* neurons (yellow bar, 30-Hz laser) in an anesthetized mouse. Inspiration (Insp), downward deflections on airflow trace; expiration (Exp), upward deflections. Note ultrasonic vocalizations of increasing volume and duration during optogenetic stimulation, accompanied by breathing changes. Insets (above), induced vocalizations expanded to millisecond timescale. Note ultrasonic vocalizations with motifs that repeat at the same rate as stimulus pulse. **c**, Airflow trace of single breaths before (top) and during (bottom) optogenetic stimulation of RAm *Nts* neurons. Note that tidal volume (V_T_, shaded area) and expiration time (T_E_) increase during optogenetic stimulation. **d**, Effect of pulse frequency on vocalizations generated by optogenetic stimulation of RAm *Nts* neurons in an anesthetized *Nts*^*Cre*^ mouse. Note lack of vocalization with 10-Hz stimulation, audible (<20 kHz) vocalizations generated with increasing volume from 15 Hz to 20 Hz and ultrasonic (>20 kHz) vocalizations generated with increasing volume from 25 Hz to 30 Hz. Amp., sound amplitude. **e**,**f**, Peak syllable amplitude of optogenetically driven vocalizations (*n* = 9 mice, colored lines) in audible (**e**) and ultrasonic (**f**) frequency ranges. Note monotonic increase in peak syllable amplitude of audible vocalizations with increasing stimulation frequency in all mice and similar monotonic increase in ultrasonic vocalization amplitude in a subset of mice that produced ultrasonic vocalizations. **g**,**h**, Syllable pitch for the same optogenetic stimulation trials as **e**,**f**. Note that syllable pitch does not change. **i**, Histograms of peak syllable amplitude of all vocalizations generated during RAm *Nts* neuron ablation experiments (Fig. 2) (*n* = 7 mice per condition and *n* = 8,310 total syllables analyzed). Note decrease in peak syllable amplitude of residual syllables after Casp3-mediated ablation of RAm *Nts* neurons (*P* = 0.02, Pre-Casp3 versus Post-Casp3). **j**, Histograms of syllable pitch for the same syllables as **i**. Note that syllable pitch does not change after RAm *Nts* ablation (*P* = 0.7 Pre-Casp3 versus Post-Cas*p*3, *n* = 7 mice per condition and *n* = 8,310 total syllables analyzed). *P < 0.05 and NS, not significant, by paired two-tailed *t-*test. Source data

**Extended Data Fig. 4:**
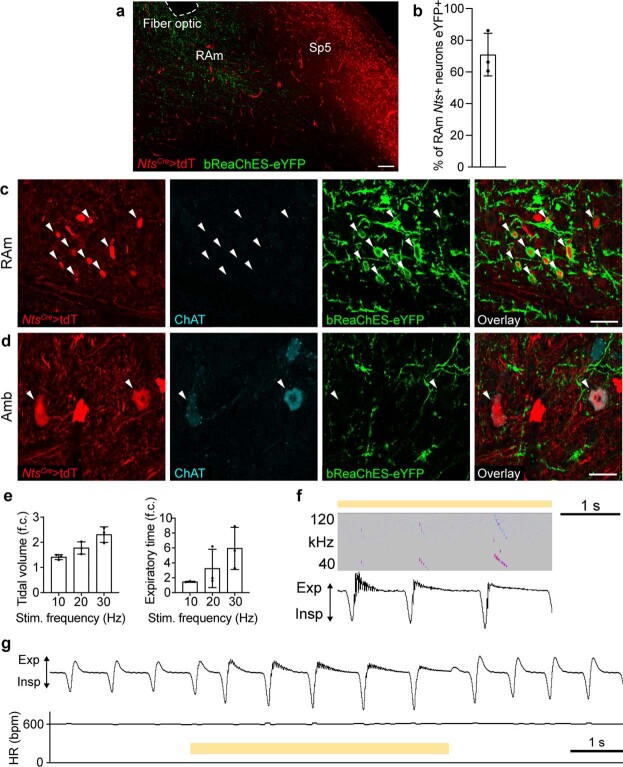
Expression of bReaChES-eYFP in RAm *Nts* neurons and stimulation-induced changes in respiration and heart rate. **a**, Sagittal section immunostaining of *Nts*^*Cre*^;*tdTomato* mouse caudal medulla following AAV-DIO-bReaChES-eYFP injection into RAm and fiber optic implantation. Note eYFP expression in RAm but not Sp5. tdTomato expression in brain vasculature (bright red streaks) was only observed after lineage labeling, presumably from transient embryonic expression since postnatal vascular *Nts* expression was not observed with *Nts* smFISH (Fig. 1) or by in situ hybridization in the Allen Brain Atlas^9^. Scale bar, 100 µm. **b**, Quantification of percent of RAm *Nts* neurons targeted by bReaChES-eYFP (*n* = 384 total *Nts*+ neurons scored in 3 mice). **c**, Immunostaining of RAm *Nts* neurons expressing bReaChES-eYFP (arrowheads) in *Nts*^*Cre*^;*tdTomato* mouse. Scale bar, 25 µm. **d**, Immunostaining of Amb *Nts*+ neurons in *Nts*^*Cre*^;*tdTomato* mouse injected with AAV-DIO-bReaChES-eYFP. Note lack of eYFP expression in ChAT+ (Amb) neurons (arrowheads) or in vasculature (bright red), indicating AAV8 approach was selective for RAm interneurons and avoided nearby motor neurons. Scale bar, 25 µm. **e**, Respiratory effects of RAm *Nts* optogenetic stimulation. Note progressive increase in tidal volume and expiratory time with increasing optogenetic stimulation frequency. f.c., fold change from pre-stimulation baseline (*n* = 3 mice). **f**, Magnified view of airflow oscillations and their temporal relationship to ultrasonic vocalizations elicited by RAm *Nts* optogenetic stimulation. Note expiratory airflow oscillations during ultrasonic vocalization production, and dampening of oscillations as syllable volume progressively increases. **g**, Respiration airflow (top) and heart rate (HR, bottom) traces during optogenetic stimulation (yellow bar, 30 Hz) of RAm *Nts* neurons as in Fig. 3. Note lack of heart rate response during optogenetic stimulation, indicating AAV8 approach and *Nts*^*Cre*^ driver was selective to RAm interneurons and avoided nearby cardiac control neurons. Source data

**Extended Data Fig. 5:**
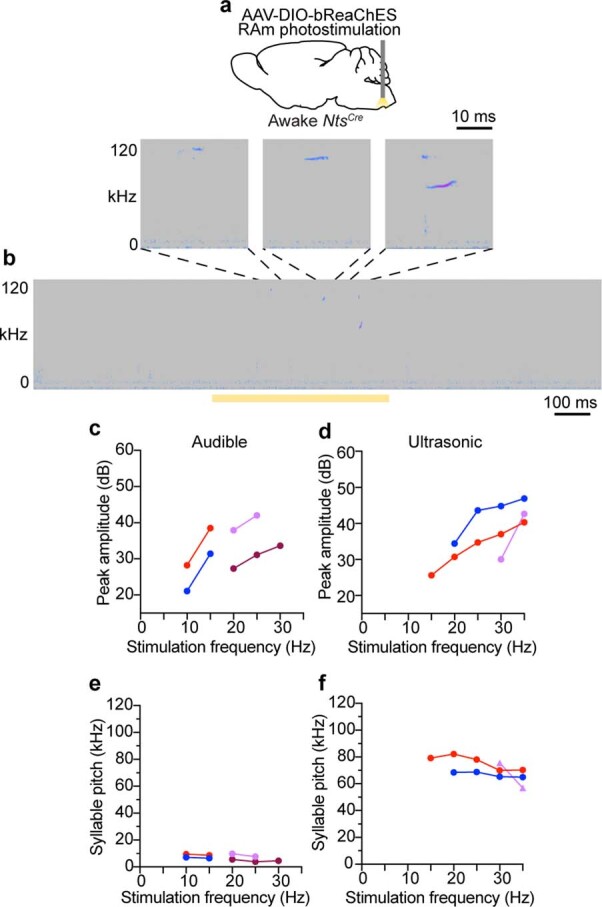
Optogenetic stimulation of RAm *Nts* neurons produces vocalization in awake mice. **a**, Schematic of optogenetic stimulation paradigm. **b**, Sonogram during optogenetic stimulation of RAm *Nts* neurons (yellow bar, 20 Hz laser light) in awake mouse. Note ultrasonic vocalizations produced within stimulation period (top, magnification). **c**, **d**, Quantification of peak syllable amplitude of optogenetically-driven vocalizations (n = 4 mice) in the audible and ultrasonic frequency ranges. Colored lines, individual mice. Note monotonic increase in all mice in peak syllable amplitude of audible vocalizations (left panel) with increasing stimulation frequency, and similar monotonic increase in ultrasonic vocalization amplitude (right panel) for mice that transitioned to ultrasonic. **e**, **f**, Quantification of syllable pitch for same audible and ultrasonic optogenetic stimulation trials as c,d. Note lack of change in syllable pitch for individual mice with increasing stimulation frequency. Source data

To determine if RAm *Nts* neuronal activity is sufficient to produce vocalization, we optogenetically activated them in anesthetized mice. A Cre-dependent AAV8 vector encoding the channelrhodopsin bReaChES was bilaterally delivered to the RAm of adult *Nts*^*Cre*^ mice, and fiber-optic cannulas were implanted above the injection sites (Fig. 3a). After AAV injection into RAm, ~70% of *Nts*^+^ neurons in the region expressed bReaChES-eYFP, and this labeling was selective for RAm *Nts*^+^ interneurons (Extended Data Fig. 4a–d). Two to four weeks after optical fiber implantation, mice were anesthetized with isoflurane and placed in a nose cone fitted with a spirometer and an ultrasonic microphone to simultaneously measure breathing and vocalization. Upon optogenetic stimulation of RAm *Nts* neurons, the anesthetized mice emitted vocalizations (Fig. 3b). They also showed related changes in breathing, as detailed below, but otherwise remained fully quiescent. During a single stimulation train, the inspiratory volume and expiratory time progressively increased with each breath (Fig. 3b,c and Extended Data Fig. 4e). Simultaneously, the airflow trace began oscillating rapidly but only during expiration, suggestive of airflow instabilities^6^ or vocal fold vibration (Fig. 3b and Extended Data Fig. 4f). During the largest breaths in the stimulation train, vocalization was produced. Although these optogenetically induced vocalizations occupied a similar fundamental frequency range as natural mouse ultrasonic vocalizations (30–110 kHz)^11^, their spectrographic form did not resemble that of any natural vocalization. Rather, the spectrogram reflected features of the laser pulses, with repeating spectral motifs time-locked to each pulse (Fig. 3b). Optogenetic stimulation did not alter heart rate (Extended Data Fig. 4g), which is powerfully regulated by neurons intermingled with RAm^14,15^, confirming that RAm *Nts* neurons had been specifically targeted. Optogenetic stimulation in awake, freely moving mice also induced vocalizations (Extended Data Fig. 5a,b). We conclude that optogenetic stimulation of RAm *Nts* neurons is sufficient to generate artificial vocalizations.

### RAm *Nts* neuronal activity controls sound volume

**Extended Data Fig. 6:**
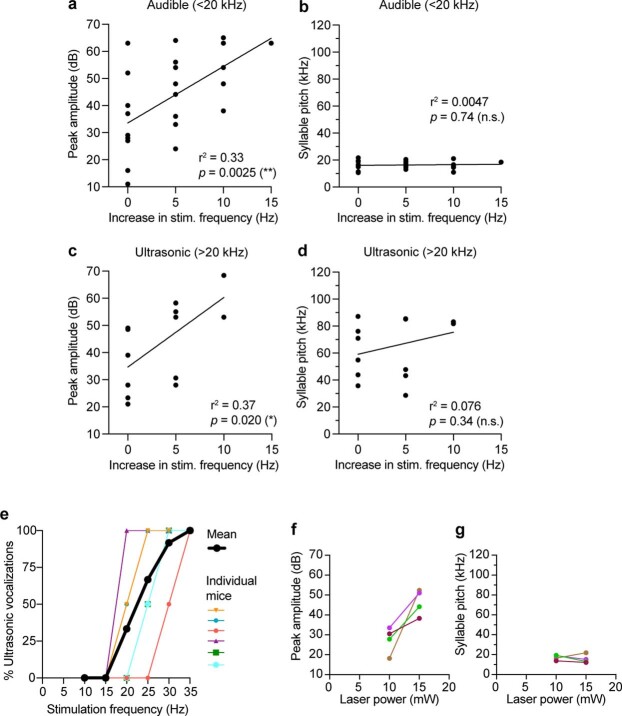
Additional quantification of relationship between optogenetic stimulation frequency and acoustic features in anesthetized mice. **a**, Relationship between RAm *Nts* optogenetic stimulation frequency and audible vocalization syllable amplitude. Data from Fig. 3e was aligned to the vocalization threshold (‘0’ on X axis indicates lowest stimulation frequency at which vocalization was elicited). Note progressive increase in peak amplitude with increasing stimulation frequency. **b**, Relationship between RAm *Nts* optogenetic stimulation frequency and audible vocalization syllable pitch for same syllables as panel a. Note lack of change in syllable pitch with increasing stimulation frequency. **c**, Relationship between RAm *Nts* optogenetic stimulation frequency and ultrasonic vocalization syllable amplitude. Data from Fig. 3f was aligned to the vocalization threshold. Note progressive increase in peak amplitude with increasing stimulation frequency. **d**, Relationship between RAm *Nts* optogenetic stimulation frequency and ultrasonic vocalization syllable pitch for same syllables as panel c. Note lack of change in syllable pitch with increasing stimulation frequency. **e**, Ultrasonic transition points of mice that transitioned from audible to ultrasonic vocalization with RAm *Nts* optogenetic stimulation. y axis indicates percent of optogenetically-driven vocalizations in the ultrasonic range (>20 kHz) at each stimulation frequency. Colored lines, individual mice (*n* = 6 mice). Black line, mean across all mice. Note most mice transitioned from audible to ultrasonic vocalization between 20 Hz and 30 Hz optogenetic stimulation. **f**, **g**, Quantification of peak syllable amplitude (left) and syllable pitch (right) of audible vocalizations elicited by RAm *Nts* optogenetic stimulation at different laser powers, while maintaining a constant pulse rate (25 Hz). Note increase in syllable loudness as laser power was increased, but lack of change in syllable pitch. Colored lines, individual mice (*n* = 4 mice). All *r*^2^ and *p* values in panels a-d calculated from simple linear regression. *: *p* < 0.05, **: *p* < 0.01, n.s.: not significant. Source data

To investigate acoustic features regulated by RAm *Nts* neuronal activity, we systematically altered the laser pulse frequency of RAm *Nts* optogenetic activation in anesthetized mice. A distinct minimum pulse frequency was required to elicit vocalization in different mice. At this threshold, low-intensity vocalizations were detected in the audible frequency range (<20 kHz fundamental frequency) (Fig. 3d,e). As pulse frequency was increased, the loudness of the audible vocalizations increased monotonically in all mice (Fig. 3e and Extended Data Fig. 6a), whereas pitch remained unchanged (Fig. 3g and Extended Data Fig. 6b). Then, above a second threshold of pulse frequency, the induced vocalizations transformed from audible to ultrasonic (>20 kHz fundamental frequency) (Fig. 3d,f and Extended Data Fig. 6e). When these ultrasonic vocalizations could be elicited with multiple pulse frequencies, their loudness also increased with pulse frequency (Fig. 3f and Extended Data Fig. 6c), and, likewise, there was no consistent change in pitch (Fig. 3h and Extended Data Fig. 6d). Similar effects were observed when laser power was increased with a constant pulse frequency (Extended Data Fig. 6f,g). Increasing the stimulation rate in awake, freely moving mice also increased loudness without altering pitch (Extended Data Fig. 5c–f). Thus, the neural activity level of RAm *Nts* neurons controls the loudness of vocalizations as well as the transition from audible to ultrasonic, but it does not regulate pitch within the audible or ultrasonic regime.

To further investigate the relationship between RAm *Nts* neurons and sound volume, we examined the few remaining syllables in the RAm *Nts* neuron-ablated animals described above. Here, too, we observed the same relationship between RAm Nts neural activity and sound volume: ablation of RAm *Nts* neurons greatly reduced the loudness of the remaining syllables relative to that of control, pre-ablation syllables (Fig. 3i), whereas syllable pitch remained unchanged (Fig. 3j). Thus, both gain-of-function and loss-of-function studies support that RAm *Nts* neurons control sound volume, and the relationship is monotonic and spans the full range from silence to loud social vocalizations.

### RAm *Nts* neurons control laryngeal and expiratory muscles

**Fig. 4:**
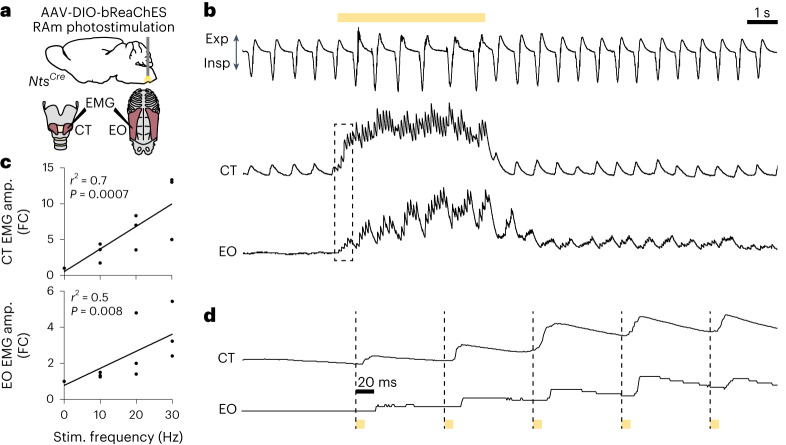
Optogenetic activation of RAm *Nts* neurons drives high-fidelity, short-latency spikes in laryngeal and expiratory muscles. **a**, Schematic of optogenetic stimulation with EMG recording. *Nts*^*Cre*^ mice were bilaterally injected with AAV-DIO-bReaChES into RAm. An optical fiber delivered yellow laser light into RAm while EMG was simultaneously recorded from laryngeal (CT) and expiratory (EO) muscles and airflow by a spirometer. **b**, Airflow trace (top), integrated amplitude EMG trace for CT (middle) and EO (bottom) during optogenetic stimulation of RAm *Nts* neurons (yellow bar, 10 Hz). Note rhythmic inspiratory activity of CT muscle and lack of EO activity during eupneic (normal) breathing before stimulus and then increases in CT and EO EMG activity during optogenetic stimulation, followed by gradual return to pre-stimulation breathing and EMG patterns. **c**, Quantification of integrated EMG amplitude (amp.) fold change (FC) from pre-stimulation period for CT (top) and EO (bottom) muscles during 0-Hz, 10-Hz, 20-Hz and 30-Hz optogenetic stimulation (*n* = 3 mice). Note progressive increase in EMG amplitude of both muscles with increasing stimulation frequency. CT: *P* = 0.0007 and EO: *P* = 0.008 by linear regression. **d**, Magnified traces from dashed boxed region in **b** during optogenetic stimulation pulses (yellow bars). Note short-latency, high-fidelity EMG spikes in both muscles after an optogenetic stimulation pulse but slightly longer latency of EO (~20 ms) versus CT (~8 ms) activation. Source data

To investigate the mechanism by which RAm *Nts* neurons produce sound and control its volume, we monitored the activity of two key vocalization muscles by electromyogram (EMG) recording while optogenetically stimulating RAm *Nts* neurons. One of the muscles was the cricothyroid (CT), a key laryngeal muscle contributing to laryngeal adduction, vocal fold tension and vocal pitch^16^. The other was the external oblique (EO), a major abdominal muscle that generates expiratory force during vocalization and contributes to sound volume^17^. RAm *Nts* neurons were optogenetically stimulated under isoflurane-anesthetized conditions as above while EMG was simultaneously recorded from both muscles (Fig. 4a). During eupneic breathing, the CT demonstrated a low level of rhythmic inspiratory activity, consistent with previous EMG studies^18^, whereas the EO remained silent, also consistent with previous work^19^. Optogenetic stimulation of RAm *Nts* neurons drove rapid and stimulation rate-dependent increases in EMG amplitude of both CT and EO (Fig. 4b,c), indicating coordinated contraction of both muscles. CT muscle activity rapidly returned to baseline levels after cessation of stimulation, whereas the EO muscle had slower off-kinetics, gradually returning to baseline voltage over several seconds (Fig. 4b). Examining the EMG traces at the millisecond timescale (Fig. 4d) revealed that single laser pulses elicited coordinated EMG spikes in the CT and EO muscles with high fidelity and low latency. However, CT had a slightly shorter latency (~8 ms) than EO (~20 ms), a difference that we explore further below. Thus, optogenetic activation of RAm *Nts* neurons drives coordinated contraction of key laryngeal and expiratory muscles involved in phonation and volume control.

### RAm *Nts* neurons innervate laryngeal and respiratory neurons

**Fig. 5:**
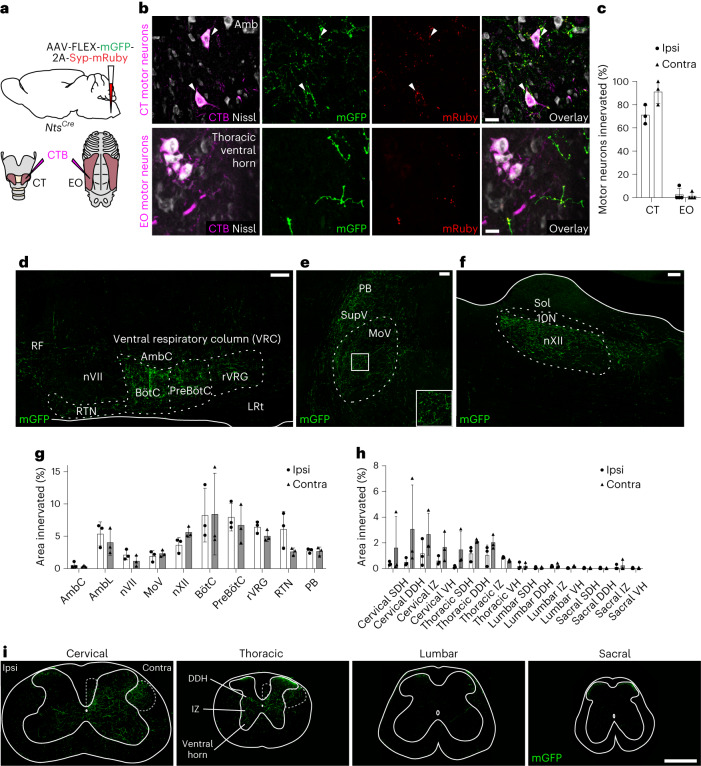
Projection targets of RAm *Nts* neurons. **a**, RAm *Nts* neuron projection mapping. *Nts*^*Cre*^ mice were injected in RAm with the AAV indicated for Cre-dependent labeling of axons (mGFP) and putative synapses (mRuby). After AAV injection, retrograde tracer CTB was injected into CT and EO muscles to label their motor neurons. **b**, Immunostaining of retrogradely labeled (CTB^+^) CT motor neurons in Amb (top) and EO motor neurons in thoracic ventral horn (VH) (bottom) in *Nts*^*Cre*^ mice injected in RAm with the AAV. RAm *Nts* neurons (mGFP^+^ and mRuby^+^) directly innervate (red puncta) CT (arrowheads) but not EO motor neurons. Scale bars, 20 μm. **c**, CT and EO motor neuron innervation by ipsilateral (left bars) and contralateral (right bars) RAm *Nts* neurons (CT: *n* = 3 mice; EO: *n* = 4 mice). **d**, Sagittal section immunostaining of contralateral ventral respiratory column (VRC) after mGFP labeling of RAm *Nts* fibers as above. Note selective projection of RAm *Nts* neurons to VRC, comprising RTN/parafacial respiratory group, BötC, pre-BötC and rVRG, plus sparse innervation of pontine reticular formation (RF). Scale bar, 100 µm. **e**, Sagittal section immunostaining of contralateral trigeminal motor nucleus (MoV) as above. Note innervation of a subset of MoV, with dense innervation of supratrigeminal nucleus (SupV) and parabrachial (PB)/Kӧlliker–Fuse nuclei. Inset, boxed region of MoV. Scale bar, 100 µm. **f**, Sagittal section immunostaining of contralateral hypoglossal motor nucleus (nXII) as above. Note innervation of nXII. 10N, dorsal motor nucleus of vagus; Sol, nucleus of the solitary tract. Scale bar, 100 µm. **g**,**h**, Quantification of mGFP^+^ innervation density of RAm *Nts* neurons across brainstem (**g**, *n* = 3 mice) and spinal cord (**h**, *n* = 3 mice) regions. DDH, deep dorsal horn; IZ, intermediate zone; SDH, superficial dorsal horn. **i**, Transverse section immunostaining of cervical, thoracic, lumbar and sacral spinal cord after mGFP labeling of RAm *Nts* axons. Note RAm axons descending contralaterally through the dorsal corticospinal tract (middle dashed outline) and lateral spinal nucleus (right dashed outline), with extensive arborization in cervical and thoracic DDH, IZ and VH. Scale bar, 500 µm. Data are shown as mean ± s.d. Contra, contralateral; Ipsi, ipsilateral. Source data

To determine if RAm *Nts* neurons innervate CT motor neurons, we labeled and mapped their axons and synapses. *Nts*^*Cre*^ mice were injected with a Cre-dependent AAV encoding mGFP and a synaptophysin–mRuby fusion protein, which labeled RAm *Nts* axons with GFP and putative pre-synaptic terminals with mRuby (Fig. 5a). CT motor neurons were retrogradely labeled by cholera toxin B (CTB) (Fig. 5a). RAm *Nts* neurons bilaterally and directly innervated almost all CT motor neurons (Fig. 5b,c), consistent with the short-latency activation observed for this muscle after optogenetic stimulation.

In addition to their innervation of CT motor neurons, ascending projections from RAm *Nts* neurons densely innervated breathing control regions across the brainstem, including the ventral respiratory column (retrotrapezoid nucleus (RTN)/parafacial respiratory group, Bötzinger complex (BӧtC), pre-Bӧtzinger complex (pre-BӧtC) and rostral ventral respiratory group (rVRG)) as well as the parabrachial/Kӧlliker–Fuse nuclei (Fig. 5d,e,g). RAm *Nts* neurons also innervated a subpopulation of trigeminal (jaw) motor neurons (Fig. 5e,g) and hypoglossal (tongue) motor neurons (Fig. 5f,g) but spared brainstem nuclei not known to be involved in vocalization, such as the nucleus ambiguus compact formation (AmbC, esophageal motor neurons) and the dorsal motor nucleus of vagus (10N) (Fig. 5d,f). Thus, ascending projections from RAm *Nts* neurons directly innervate the breathing and orofacial motor regions involved in phonation.

### RAm *Nts* neurons project to spinal cord expiratory centers

To determine the spinal cord projection targets of RAm *Nts* neurons, we labeled their axons and synapses with mGFP and mRuby as above and mapped their projections in the spinal cord. RAm *Nts* axons primarily descended contralaterally along two distinct spinal cord tracts: the dorsal corticospinal tract and a second tract in the lateral spinal nucleus (Fig. 5i). At the cervical and thoracic levels, which control breathing muscles, axons arborized and innervated spinal gray matter across the deep dorsal horn, intermediate zone and ventral horn (Fig. 5h,i), a distribution similar to abdominal muscle pre-motor neuron distributions^20^. Unlike classical studies of RAm innervation using bulk tracing methods^5^, we found that RAm *Nts* innervation declined precipitously at the lumbar and sacral levels (Fig. 5h,i), which control hindlimb and pelvic muscles.

To determine if RAm *Nts* neurons directly innervate EO motor neurons, we retrogradely labeled EO motor neurons with CTB. In contrast to CT motor neurons, RAm *Nts* neurons rarely innervated EO motor neurons (<10%) (Fig. 5b,c). However, they extensively innervated nearby interneurons, suggesting indirect innervation involving local microcircuits, consistent with the longer latency activation and slower off-kinetics observed above for the EO muscle after RAm *Nts* optogenetic stimulation. We conclude that RAm *Nts* neurons send descending projections to spinal cord segments controlling expiratory muscles, but, unlike their direct projection to CT motor neurons, they project indirectly to EO motor neurons.

## Discussion

**Fig. 6:**
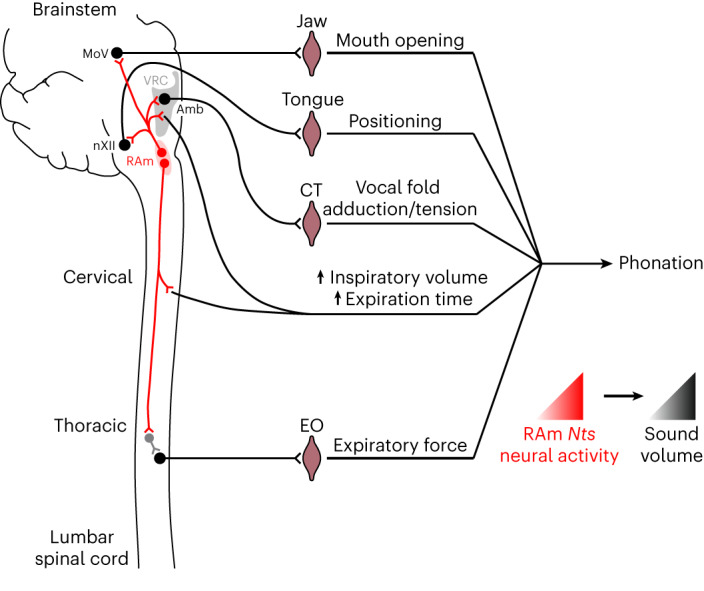
Summary of RAm *Nts* neuron innervation and function. RAm *Nts* neurons (red) innervate the trigeminal motor nucleus (MoV), which contains jaw opening motor neurons; the ventral respiratory column (VRC) and cervical spinal cord, which increase inspiratory (tidal) volume and expiratory time; Amb CT motor neurons, which adduct and tense the vocal folds; and the hypoglossal nucleus (nXII), which positions the tongue. RAm *Nts* neurons generate expiratory force through their indirect innervation of EO motor neurons in the spinal cord, possibly through a local interneuron (gray). All the above functions combine to produce sound (phonation). In addition to generating phonation, the RAm *Nts* neuronal activity level (red gradient triangle) determines the volume of the produced sound (black gradient triangle) by adjusting abdominal expiratory pressure via the EO muscle. RAm *Nts* neurons receive input from the midbrain periaqueductal gray and probably from other brain regions (not shown) to initiate phonation and adjust volume during diverse vocalizations.

We identified and characterized RAm *Nts* neurons, revealing a neural circuit for phonation (Fig. 6). RAm *Nts* neurons are an excitatory subpopulation of ~160 neurons located near the spinomedullary junction, an ideal position to coordinate phonation. They are robustly activated by both neonatal isolation cries (aversive context) and adult social vocalizations (appetitive context), showing that they are involved in a broad range of, and potentially all, vocalizations. Ablation of RAm *Nts* neurons in adult mice abolishes adult male-to-female social vocalizations, whereas optogenetic activation generates artificial vocalizations that reflect features of the stimulus. RAm *Nts* neurons produce sound by recruiting two key vocalization muscles: the CT, which adducts/tenses the vocal folds and is recruited through direct RAm *Nts* projections, and the EO, which generates expiratory force and is recruited through indirect projections. The indirect projection to EO provides a ~12-ms delay in activation compared to CT, ensuring that the vocal folds are fully adducted and tensed before subglottal pressure is generated by the EO. In addition to the CT and EO projections, RAm *Nts* neurons project to all breathing and orofacial nuclei that generate the other key components of phonation: mouth opening (MoV), tongue positioning (nXII), increased tidal volume (pre-BӧtC, rVRG and cervical spinal cord) and increased expiration time (BӧtC and RTN). Thus, the RAm *Nts* circuit appears to be the core neural circuit for phonation.

In addition to phonation, RAm *Nts* neurons also control a key acoustic feature of vocalization: sound volume. Gain-of-function and loss-of-function studies show that RAm *Nts* neural activity monotonically determines the volume of the produced sound at audible and ultrasonic frequencies, spanning the full range from silence to loud social vocalizations. Our respiration and EMG data suggest a simple mechanism by which RAm *Nts* neurons increase sound volume. Increasing RAm *Nts* neuronal activity increases tidal volume, which increases the elastic recoil pressure of the lungs and vocal loudness^17^. Increasing RAm *Nts* neuronal activity also increases activity of the EO muscle, a muscle known to increase loudness by increasing expiratory force and subglottal pressure^17^. Our results are in agreement with a recent single-unit recording study in the rat RAm, which identified a subpopulation of RAm neurons with a tonic activity pattern that was highly correlated with vocal loudness^21^. Our study also complements a recent study that showed that hypothalamic lateral preoptic area neurons expressing estrogen receptor 1 can scale the volume and bout length of adult male social vocalizations^22^. RAm *Nts* neurons may provide a downstream or parallel mechanism to adjust sound volume, because they are activated in multiple vocalization contexts.

RAm *Nts* neuronal activity also controls the transition from audible to ultrasonic vocalization. Rodent ultrasonic vocalizations are thought to be produced by a whistle mechanism generated by a glottal air jet impinging on the thyroid inner wall^23^. Critical levels of laryngeal adduction and subglottal pressure are necessary to generate ultrasonic vocalization through this mechanism^23^. Our data suggest that a critical level of RAm *Nts* neuronal activity is necessary to reach threshold laryngeal adduction and subglottal pressure levels and convert audible vocalizations to ultrasonic.

How does the brain control the other acoustic features of innate vocalization, such as pitch, syllable structure and syntax? These features are thought to be controlled within the pons and medulla, where neurons tuned to some of these features have been identified in multiple regions, including RAm^24,25^. Artificial stimulation of the PAG generates natural-sounding vocalizations^3^, consistent with acoustic features being controlled by downstream structures. In contrast, RAm *Nts* neuron stimulation produced artificial vocalizations, suggesting that cell types downstream of PAG in addition to RAm *Nts* neurons are required to generate natural vocalizations. Notably, RAm *Nts* neurons comprise only ~45% of the vocalization-activated RAm neurons, and some of the other neurons are also directly innervated by PAG. Thus, there is at least one other RAm vocalization subpopulation. These might control acoustic features other than volume and may be necessary along with RAm *Nts* neurons to create natural-sounding vocalizations.

The function of RAm appears to be conserved across vocalizing vertebrates^4,24,26^, suggesting that the RAm *Nts* circuit may also be conserved. RAm is present in the avian brain and is thought to be the final common pathway for both innate calls and learned songs^27^. The avian RAm possesses a remarkably similar circuit architecture to the mammalian RAm *Nts* circuit, containing pre-motor neurons that innervate the vocal motor neurons, abdominal expiratory neurons and breathing control nuclei^28^. Additionally, RAm is present in all mammalian species examined, including rodents, non-human primates^24^ and humans^29^. As was found in our study, studies in primates have found that RAm contains a dense concentration of neurons activated by diverse innate vocalizations^24^. Hence, it is possible that RAm mediates human innate vocalizations, such as laughter and crying, and it will be important to determine if it harbors a homolog of the mouse *Nts* circuit that provides the core phonation drive for these vocalizations. Projections from human motor cortex innervate the RAm region as well as Amb^30^, suggesting that RAm may be engaged to generate phonation during human speech while parallel human-specific projections to Amb^31^ generate fine pitch adjustments. Similar circuit architectures that use brainstem pre-motor modules have been identified in other skilled motor control circuits^32^. Engagement of the RAm *Nts* module to generate the basic phonation drive for diverse vocalizations while controlling their volume would explain the strong conservation of RAm across vocalizing species.

## Methods

### Mice

Wild-type C57BL/6NCrl mice (Charles River Laboratories, strain 027), *Nts*^*Cre*^ knock-in mice^34^ (Jackson Laboratory, strain 017525) and Ai9 or Ai14 tdTomato mice^35^ (Jackson Laboratory, strain 007909) were housed and bred in the animal facility at Stanford University in accordance with Institutional Animal Care and Use Committee (IACUC) guidance and were maintained on a 12-h light/dark cycle at temperature 70–75 °F and humidity 35–60%, with food and water provided ad libitum. Male mice of age 6–8 weeks were used for all adult social vocalization studies. Both male and female mice of age 6–8 weeks were used for all optogenetic stimulation and viral tracing studies. Neonatal mice were postnatal day 7 and were not sexed. All mouse experiments were approved by the Stanford University IACUC.

### Vocalization induction for neural activity monitoring by *Fos* labeling

For adult social vocalization induction, adult (6–8 weeks old) male wild-type C57BL/6NCrl mice were individually housed 1 d before experiments. The next day, an adult wild-type female was placed in the male’s cage, and an ultrasonic microphone (Avisoft Bioacoustics, CM16/CMPA) was used to verify vocalization production. After 90 min, the male was immediately killed by CO_2_ inhalation and transcardial perfusion for smFISH studies.

For neonatal isolation cry induction, postnatal day 7 wild-type mice were removed from their home cage and placed in a large plastic box while an ultrasonic microphone was used to verify vocalization production. After 90 min, the pup was immediately killed by saturating vapors of isoflurane and transcardial perfusion for smFISH studies.

### AAV injection and optical fiber implantation

Adult *Nts*^*Cre*^ mice (6–8 weeks old) were anesthetized with isoflurane (3% for induction and 1–2% for maintenance) for AAV injections. Anesthetized mice were placed in a stereotactic instrument (David Kopf Instruments, model 940) with body temperature maintained at 37 °C using a feedback-controlled heating pad (Physitemp Instruments, TCAT-2LV). Immediately before surgery, mice were given analgesic (carprofen 5 mg kg^−1^ and buprenorphine SR 0.5–1.0 mg kg^−1^, subcutaneous). The following AAV vectors were used: for caudolateral PAG labeling, AAVDJ-CAG-GFP (9.3 × 10^12^ genome copies per milliliter (GC ml^−1^), Stanford Gene Vector and Virus Core); for cell ablation, AAV8-Ef1a-FLEX-taCasp3-TEVp^12^ (8.8 × 10^13^ GC ml^−1^, Addgene, 45580, Janelia Viral Tools facility); for mock ablation, AAV8-CAG-FLEX-GFP (UNC Vector Core); for optogenetic stimulation, AAV8-Ef1a-DIO-bReaChES-TS-eYFP (2.9 × 10^12^ GC ml^−1^, Stanford Gene Vector and Virus Core); and for projection mapping, AAVDJ-hSyn-FLEX-mGFP-2A-Synaptophysin-mRuby^36^ (1.2 × 10^13^ GC ml^−1^, Stanford Gene Vector and Virus Core). To target the caudolateral PAG, 50 nl of AAV-GFP was unilaterally injected on the left side at the following stereotaxic coordinates: −4.5 mm caudal to bregma, −0.7 mm lateral and −2.0 mm ventral to the surface of the brain. To target RAm in *Nts*^*Cre*^ mice, 500–700 nl of the GFP, Casp3 or bReaChES AAV vector was bilaterally injected at the following stereotactic coordinates: 3.4 mm caudal to lambda, ±1.25 mm lateral to lambda and 6.3 mm ventral to lambda. Immediately after bilateral AAV-DIO-bReaChES injection, fiber-optic cannulas (Doric Lenses, MFC_200/230-0.37_6mm_ZF1.25_FLT) were bilaterally implanted 350 μm above the injection site and secured to the skull with dental cement (Parkell, C&B Metabond). For all experiments, mice in which each injection did not target RAm on histology were excluded from analysis. For projection mapping experiments, 100 nl of the Syp-mRuby AAV vector was injected unilaterally into the left RAm. Mice recovered for 4 weeks for ablation experiments and 3–8 weeks for optogenetic or projection mapping experiments.

### Adult vocalization recordings in neural ablation experiments

For vocalization recording before ablation, adult male *Nts*^*Cre*^ mice (6–8 weeks old) were individually housed 1 d before recording. On the day of recording, the male’s cage was placed in a black plastic box with an ultrasonic microphone (Avisoft Bioacoustics, CM16/CMPA) and a video camera mounted above the cage. After 15 min of acclimation time, an adult wild-type female was placed in the male’s cage for 5 min, and the encounter was recorded. Female mice were verified to be in estrus^37^ on the day of recording to maintain consistency between trials. The next day, male *Nts*^*Cre*^ mice were randomized to ablation or control groups, and AAV8-Ef1a-FLEX-taCasp3-TEVp or AAV8-CAG-FLEX-GFP (mock ablation control) was bilaterally injected into RAm. After a 4-week recovery to allow for protein expression and cell ablation, vocalization was recorded as above in a 5-min encounter with a wild-type female in estrus. The male was then killed by transcardial perfusion. Vocalizations were assumed to be produced by the male because males produce the majority of vocalizations while in the presence of a female^38^.

### Ultrasonic vocalization and behavior analysis

Adult social vocalization sound files were analyzed using MATLAB (MathWorks) with MUPET^39^. To extract syllables, sound files were processed using default MUPET parameters, with the exception of the following: minimum-syllable-duration, 8; minimum-syllable-total-energy, −40; minimum-syllable-peak-amplitude, −40; minimum-syllable-distance, 10; and minimum-usv-frequency, 50,000. All extracted syllables were manually examined by a blinded experimenter, and any falsely detected syllables due to noise from audible mouse movement were excluded from analysis. Peak syllable amplitude and syllable pitch (‘mean frequency’ in MUPET) were extracted directly from the MUPET output file. To calculate social interaction time, videos were manually scored by counting the number of seconds during the 5-min trial in which the male mouse’s nose or forelimb was in contact with the female.

### Optogenetic stimulation with recording of vocalization

*Nts*^*Cre*^ mice injected with AAV8-Ef1a-DIO-bReaChES-TS-eYFP and recovered as above were anesthetized with isoflurane (3% induction and 1–2% maintenance), and body temperature was maintained at 37 °C. Respiration was recorded using a spirometer (ADInstruments) connected to a plastic nose cone that also delivered maintenance isoflurane. Vocalizations were recorded with an ultrasonic microphone (Avisoft Bioacoustics, 40007) attached to the nose cone and connected to a Avisoft Bioacoustics UltraSoundGate 116H recording interface. Single-lead ECG was recorded using needle electrodes (ADInstruments, MLA1203), an ADInstruments Octal Bio Amp and an ADInstruments PowerLab data acquisition system. The implanted fiber-optic cannulas were connected via fiber-optic cable (Doric Lenses, SBP(2)_200/220/900-0.37_1m_SMA-2xZF1.25) to a 577-nm laser (CNI Laser), and laser light was delivered using the following parameters: 10–15-mW power from the fiber tip, 10-ms pulse width and 5-s stimulation train duration. The interval between stimulation trains was 5 min, and two stimulation trains were performed at each pulse rate. Pulse width was not varied. Laser power and pulse frequency were varied as indicated in each figure. Because of the high spike fidelity of bReaChES^40^, we assume that increasing pulse frequency increased the firing rate of RAm *Nts* neurons.

For optogenetic stimulation in awake mice, stimulation was performed as above while mice were placed in a black plastic box with an ultrasonic microphone (Avisoft Bioacoustics, CM16/CMPA) and a video camera mounted above the cage. Lower laser powers and stimulus durations were sufficient to elicit vocalization in awake mice, so the stimulation parameters were modified to a stimulus train duration of 500 ms and laser power of 5 mW.

To calculate peak syllable amplitude and syllable pitch of optogenetically driven vocalizations, sound files were analyzed using Audacity (https://www.audacityteam.org/). The onset and offset time of each syllable was manually annotated. The ‘plot spectrum’ function was applied to each syllable to calculate the fast Fourier transform. To calculate peak syllable amplitude, the peak amplitude of each syllable in dB was subtracted from the dB of quiet background noise. The frequency of the syllable at its peak amplitude was reported as the syllable pitch. When multiple syllables were recorded at a given stimulation parameter set, the loudest syllable was used to calculate the peak syllable amplitude and syllable pitch.

### Optogenetic stimulation with EO and CT muscle EMG recording

*Nts*^*Cre*^ mice injected with AAV8-Ef1a-DIO-bReaChES-TS-eYFP and recovered as above were anesthetized with isoflurane (3% induction and 1–2% maintenance), and body temperature was maintained at 37 °C. Respiration was recorded using a spirometer (ADInstruments) connected to a plastic nose cone that also delivered maintenance isoflurane. The mouse was placed in the supine position, and the skin overlying the EO muscle was aseptically prepared. A 1-cm skin incision was made to expose the EO muscle, and a two-lead needle electrode (ADInstruments, MLA1203) was inserted into the muscle. The CT muscle was similarly exposed with a 1-cm ventral neck skin incision, followed by dissection of the overlying strap muscles, and two 76.2-μm-diameter silver wires (A-M Systems) were inserted into the CT muscle. Electrodes were connected to an ADInstruments Octal Bio Amp and an ADInstruments PowerLab data acquisition system that recorded EMG and respiration at a sampling rate of 1 kHz. EMG signals were high-pass filtered at 100 Hz and then integrated using LabChart parameters: Integral, absolute value; Time constant decay, 0.2 s. Fold change of integrated EMG amplitude was calculated by dividing the peak integrated EMG amplitude during laser stimulation by the integrated EMG amplitude immediately before laser stimulation. Laser light (577 nm) was delivered as above using the following parameters: 10–15-mW power from the fiber tip and 10-ms pulse width.

### CT and EO muscle injections for RAm *Nts* neuron projection mapping

For CT and EO muscle injections, adult (age 6–8 weeks) *Nts*^*Cre*^ mice were used that had been previously injected as described above with AAVDJ-hSyn-FLEX-mGFP-2A-Synaptophysin-mRuby into RAm 8 weeks before the muscle injections. Mice were anesthetized with isoflurane (3% for induction and 1–2% for maintenance) and then pre-treated with analgesic (carprofen 5 mg kg^−1^ and buprenorphine SR 0.5–1.0 mg kg^−1^, subcutaneous). For CT injections, a 1-cm incision was made in the ventral neck, and the CT muscles were exposed by dissection of the overlying strap muscles. A pulled glass micropipette (Drummond Scientific, 5-000-2005) was then used to inject 200–300 nl of 1% CTB solution (Sigma-Aldrich, C9903, diluted in PBS + 0.05% Fast Green dye) into the left and right CT muscles. The overlying skin was sutured, and the mouse was placed in a heated recovery cage. For EO injections, 400–1,000 nl of 1% CTB was similarly injected into the left and right EO muscles through an incision in the overlying skin that was sutured after injection. Mice recovered for 3 d (CT) or 7 d (EO) before perfusion and immunostaining.

### smFISH and immunostaining

Mice were killed with CO_2_ and transcardially perfused with 4% paraformaldehyde (PFA), and tissues were post-fixed in 4% PFA overnight at 4 °C. Brains and spinal cords were cryoprotected in 30% sucrose at 4 °C overnight. Cryoprotected tissue was embedded in optimal cutting temperature (OCT) compound and sectioned on a Leica CM3050S cryostat at 20 μm for smFISH and 25 μm for immunostaining.

For smFISH, sections were processed with an RNAscope Multiplex Fluorescent Assay v2 kit according to manufacturer instructions and using the following probes: Mm-Fos (316921), Mm-Nts-C2 (420441-C2), Mm-Slc17a6-C3 (319171-C3) and Mm-Slc18a3-C3 (448771-C3).

For immunostaining, sections were permeabilized in PBS + 0.3% Triton X-100, blocked for 1 h in block buffer (PBS + 0.3% Triton + 10% normal donkey serum) and incubated with primary antibodies in block buffer at 4 °C overnight. Slides were washed three times, incubated in secondary antibodies in block buffer for 1 h at room temperature and washed three times, and a coverslip was applied with ProLong Gold Antifade Reagent. Primary antibodies included: chicken anti-GFP (Aves Labs, GFP-1010, 1:1,000), rabbit anti-c-Fos (Synaptic Systems, 226 003, 1:5,000), goat anti-ChAT (Millipore, AB144P, 1:100) and goat anti-CTB (List Labs, 703, 1:1,000). Species-specific donkey secondary antibodies conjugated to Alexa Fluor 488, 568 or 647 were obtained from Life Technologies or Jackson ImmunoResearch and used at a 1:500 dilution.

To determine the total number of RAm neurons, the cluster of c-Fos^+^ neurons after vocalization was used to define the boundaries of the region, and NeuroTrace (Invitrogen, N21479) was used to count the number of neurons within those boundaries. To count RAm *Nts* neurons in AAV experiments, the *Nts* smFISH probe or an *Nts*^*Cre*^-driven tdTomato allele was used. NeuroTrace was used to differentiate *Nts*^+^ neurons from lineage-labeled vasculature in *Nts*^*Cre*^;tdTomato mice. Stained neurons were counted manually from *z*-stacks acquired on a Zeiss LSM 780 confocal microscope. To quantify innervated RAm neurons or CT/EO motor neurons, a neuron was scored as innervated if it had at least two GFP^+^ (PAG experiment) or mRuby^+^ (CT/EO experiment) puncta directly abutting the cell soma.

To quantify RAm Nts innervation of brainstem and spinal cord nuclei, mGFP^+^ fibers were quantified in ImageJ (Fiji). ROIs were drawn around the brain regions, and the GFP channel was converted to a mask and then binarized. ‘Area fraction’ was then quantified for each ROI to calculate the percent area innervated.

### Data collection and statistics

All results are presented as mean ± s.d. with all data points displayed. All statistical analyses were performed with GraphPad Prism. All statistical tests used are listed in the figure legends, with statistical significance set at *P* < 0.05. Statistical tests were not used to pre-determine sample size, but sample sizes are similar to those in previous publications^3,22^. Data distribution was assumed to be normal, but this was not formally tested. Fos labeling and social interaction time were quantified by a blinded experimenter. Syllable count, syllable amplitude and EMG amplitude were quantified using the same automated approaches for all mice, so blinding was not relevant. Wild-type mice were randomized into control or vocalization groups for Fos labeling. Male *Nts*^*Cre*^ mice were randomized to ablation or control groups.

### Reporting summary

Further information on research design is available in the Nature Portfolio Reporting Summary linked to this article.

## Online content

Any methods, additional references, Nature Portfolio reporting summaries, source data, extended data, supplementary information, acknowledgements, peer review information; details of author contributions and competing interests; and statements of data and code availability are available at 10.1038/s41593-023-01478-2.

### Supplementary information

Reporting Summary

Supplementary Video 1 Male *Nts*^*Cre*^ mouse behavior toward female after bilateral AAV-FLEX-GFP injection into RAm (mock ablation). Note male (larger mouse) chasing female with frequent olfactory investigation.

Supplementary Video 2 Male *Nts*^*Cre*^ mouse behavior toward female after bilateral AAV-FLEX-Casp3 injection into RAm (*Nts* neuron ablation). Note male (larger mouse) chasing female with frequent olfactory investigation, similarly to GFP mock ablation condition.

### Source data

Source Data Fig. 1 Statistical source data.

Source Data Fig. 2 Statistical source data.

Source Data Fig. 3 Statistical source data.

Source Data Fig. 4 Statistical source data.

Source Data Fig. 5 Statistical source data.

Source Data Extended Data Fig. 2 Statistical source data.

Source Data Extended Data Fig. 3 Statistical source data.

Source Data Extended Data Fig. 4 Statistical source data.

Source Data Extended Data Fig. 5 Statistical source data.

Source Data Extended Data Fig. 6 Statistical source data.

## Data Availability

Source data are provided with this paper.

## Code availability

This study did not use custom software code.
