# Supplementary material for: A brainstem circuit for phonation and volume control in mice
Source: Nat Neurosci. 2023 Nov 23;26(12):2122–30. doi: 10.1038/s41593-023-01478-2 (PMC10689238; doi:10.1038/s41593-023-01478-2)
Supplement: Supplementary file 1 — Reporting Summary [file 41593_2023_1478_MOESM1_ESM.pdf]

nature portfolio

Corresponding author(s): Mark A. Krasnow

Last updated by author(s): Aug 24, 2023

Reporting Summary

Nature Portfolio wishes to improve the reproducibility of the work that we publish. This form provides structure for consistency and transparency in reporting. For further information on Nature Portfolio policies, see our Editorial Policies and the Editorial Policy Checklist.

Statistics

For all statistical analyses, confirm that the following items are present in the figure legend, table legend, main text, or Methods section.

n/a	Confirmed	
☐	☒	The exact sample size (*n*) for each experimental group/condition, given as a discrete number and unit of measurement
☐	☒	A statement on whether measurements were taken from distinct samples or whether the same sample was measured repeatedly
☐	☒	The statistical test(s) used AND whether they are one- or two-sided<br>*Only common tests should be described solely by name; describe more complex techniques in the Methods section.*
☒	☐	A description of all covariates tested
☐	☒	A description of any assumptions or corrections, such as tests of normality and adjustment for multiple comparisons
☐	☒	A full description of the statistical parameters including central tendency (e.g. means) or other basic estimates (e.g. regression coefficient) AND variation (e.g. standard deviation) or associated estimates of uncertainty (e.g. confidence intervals)
☐	☒	For null hypothesis testing, the test statistic (e.g. *F*, *t*, *r*) with confidence intervals, effect sizes, degrees of freedom and *P* value noted<br>*Give P values as exact values whenever suitable.*
☒	☐	For Bayesian analysis, information on the choice of priors and Markov chain Monte Carlo settings
☒	☐	For hierarchical and complex designs, identification of the appropriate level for tests and full reporting of outcomes
☐	☒	Estimates of effect sizes (e.g. Cohen's *d*, Pearson's *r*), indicating how they were calculated

Our web collection on statistics for biologists contains articles on many of the points above.

Software and code

Policy information about availability of computer code

Data collection	ADInstruments LabChart 8, Carl Zeiss Zen 2.3 SP1
Data analysis	NIH ImageJ (Fiji) v2.1.0, GraphPad Prism 8, ADInstruments LabChart 8, Audacity v3.2.5, MATLAB R2021b, MUPET v2.1 (https://github.com/mvansegbroeck/mupet)

For manuscripts utilizing custom algorithms or software that are central to the research but not yet described in published literature, software must be made available to editors and reviewers. We strongly encourage code deposition in a community repository (e.g. GitHub). See the Nature Portfolio guidelines for submitting code & software for further information.

Data

Policy information about availability of data

All manuscripts must include a data availability statement. This statement should provide the following information, where applicable:

- Accession codes, unique identifiers, or web links for publicly available datasets
- A description of any restrictions on data availability
- For clinical datasets or third party data, please ensure that the statement adheres to our policy

Source data for all figures are available with the paper.

Research involving human participants, their data, or biological material

Policy information about studies with human participants or human data. See also policy information about sex, gender (identity/presentation), and sexual orientation and race, ethnicity and racism.

Reporting on sex and gender	N/A
Reporting on race, ethnicity, or other socially relevant groupings	N/A
Population characteristics	N/A
Recruitment	N/A
Ethics oversight	N/A

Note that full information on the approval of the study protocol must also be provided in the manuscript.

Field-specific reporting

Please select the one below that is the best fit for your research. If you are not sure, read the appropriate sections before making your selection.

☒ Life sciences ☐ Behavioural & social sciences ☐ Ecological, evolutionary & environmental sciences

For a reference copy of the document with all sections, see nature.com/documents/nr-reporting-summary-flat.pdf

Life sciences study design

All studies must disclose on these points even when the disclosure is negative.

Sample size	Sample sizes were determined based on previous studies in the field (e.g. Tschida et al., Neuron 2019; Chen et al, Nature 2021), no statistical method was used to predetermine sample size.
Data exclusions	Criteria for animal exclusion were pre-established. Mice were excluded if the AAV injection site was not within RAm on histology.
Replication	All experiments were successfully reproduced across at least 2 independent cohorts of mice.
Randomization	Wild-type mice were randomized into control or vocalization groups for Fos labeling. Male Nts-Cre mice were randomized to ablation or control groups.
Blinding	Fos labeling and social interaction time were quantified by a blinded experimenter. Syllable count, acoustic features, and EMG amplitude were quantified using the same automated approaches for all mice so blinding was not relevant.

Reporting for specific materials, systems and methods

We require information from authors about some types of materials, experimental systems and methods used in many studies. Here, indicate whether each material, system or method listed is relevant to your study. If you are not sure if a list item applies to your research, read the appropriate section before selecting a response.

Materials & experimental systems

n/a	Involved in the study	
☐	☒	Antibodies
☒	☐	Eukaryotic cell lines
☒	☐	Palaeontology and archaeology
☐	☒	Animals and other organisms
☒	☐	Clinical data
☒	☐	Dual use research of concern
☒	☐	Plants

Methods

n/a	Involved in the study	
☒	☐	ChIP-seq
☒	☐	Flow cytometry
☒	☐	MRI-based neuroimaging

Antibodies

Antibodies used	Primary antibodies were: chicken anti-GFP (Aves Labs GFP-1010, 1:1000), goat anti-ChAT (Millipore AB144P, 1:100), goat anti-CTB (List Labs #703, 1:1000), rabbit anti-c-Fos (Synaptic Systems 226 003, 1:5,000). Secondary antibodies were: Alexa Fluor 488

	donkey anti-chicken (Jackson ImmunoResearch 703-545-155), Alexa Fluor 647 donkey anti-goat (Invitrogen A-21447), Alexa Fluor 647 donkey anti-rabbit (Invitrogen A-31573).
Validation	Aves Labs GFP-1010: 735 Citations (https://www.aveslabs.com/products/anti-green-fluorescent-protein-antibody-gfp)<br>Millipore AB144P: Antibody detects level of ChAT and has been published and validated for use in IH(P), IC, IH and WB. (https://www.emdmillipore.com/US/en/product/Anti-Choline-Acetyltransferase-Antibody,MM_NF-AB144P)<br>List Labs #703: >100 citations (https://listlabs.com/products/antibodies/?sub=anti-cholera-b-subunit-goat)<br>Synaptic Systems 226 003: validated in prior studies e.g. Veerakumar et al., Nature 2022

Animals and other research organisms

Policy information about studies involving animals; ARRIVE guidelines recommended for reporting animal research, and Sex and Gender in Research

Laboratory animals	Wild-type C57BL/6NCrl mice (Charles River, strain 027), Nts-Cre knock-in mice (Jackson Laboratory, strain #017525) and Ai9 tdTomato mice (Jackson Laboratory, strain #007909) were used. Age of mice at the start of experiments was postnatal day 7 for neonatal mice and 6-8 weeks for adult mice.
Wild animals	No wild animals were used.
Reporting on sex	Both male and female mice were used for all experiments, except when recording adult male social vocalizations which are sex-specific.
Field-collected samples	No field samples were collected.
Ethics oversight	All mouse experiments were approved by the Stanford University Institutional Animal Care and Use Committee.

Note that full information on the approval of the study protocol must also be provided in the manuscript.
